# *Herpes *zoster and the risk of ischemic and hemorrhagic stroke: A systematic review and meta-analysis

**DOI:** 10.1371/journal.pone.0171182

**Published:** 2017-02-08

**Authors:** Ying Lian, Yun Zhu, Fang Tang, Bing Yang, Ruisheng Duan

**Affiliations:** 1 Department of case administration, Qianfoshan Hospital Affiliated to Shandong University, Jinan, China; 2 Department of Oro-maxillofacial Head and Neck oncology, Ninth People’s Hospital College of Stomatology, Shanghai Jiao Tong University School of Medicine, Shanghai, China; 3 Health Management Center, Qianfoshan Hospital Affiliated to Shandong University, Jinan, China; 4 Department of Neurology, Qianfoshan Hospital Affiliated to Shandong University, Jinan, China; University of California Riverside, UNITED STATES

## Abstract

**Background:**

Herpes zoster infection and stroke are highly prevalent in the general population; however, reports have presented inconsistent findings regarding the relationship between herpes zoster infection and stroke. In this meta-analysis, we aimed to clarify this association.

**Material and methods:**

The PubMed and Embase databases were searched for studies published from their inception to January 2016. Two investigators independently extracted the data. The pooled relative risk (RR) was calculated using a random effects model.

**Results:**

A total of 8 studies met the inclusion criteria. During the first 1 month after herpes zoster infection, the pooled RRs for ischemic stroke and hemorrhagic stroke were 1.55 (95% CI, 1.46–1.65) and 1.70 (95% CI, 0.73–3.96), respectively, and within 3 months after infection, the corresponding RRs were 1.17 (95% CI, 1.12–1.23) and 2.05 (95% CI, 1.17–3.60), respectively. At 1 year and more than 1 year after herpes zoster infection, a significant relationship was not observed between herpes zoster infection and the incidence of ischemic and hemorrhagic stroke. Publication bias was not observed.

**Conclusion:**

The accumulated evidence generated from this systematic review indicates that an increased risk for ischemic stroke occurred in the short term after herpes zoster infection, whereas a significant relationship was not observed in the long term after infection. With respect to hemorrhagic stroke, the association was not significant. With respect to hemorrhagic stroke, the association between was not significant except within 3 months after a herpes zoster infection.

## Introduction

Herpes zoster (HZ) infectious outbreaks, also called shingles, are caused by the reactivation of the varicella-zoster virus (VZV). Primary infection with VZV in childhood manifests as chickenpox, and then VZV enters a dormant period in the dorsal root ganglia. After VZV reactivates, it travels along sensory nerve endings and causes neuronal damage to the corresponding dermatome of the skin, where it is characterized by a vesicular rash [1,2]. Spontaneous reactivation of VZV may occur in the elderly and individuals with compromised cell-mediated immunity; therefore, the risk of an HZ outbreak substantially increases with age and immunosuppression. Accumulating evidence has shown that more than 95% of adults worldwide are infected with VZV, and approximately 30% will develop HZ in their lifetime, with this proportion increasing to 50% in those aged at least 85 years [3,4].

Stroke is one of the leading causes of deaths and disability throughout the world [5,6], and it is a multifactorial disease resulting from interactions between many risk factors. Previous studies have found that infection appears to be an important trigger that precedes up to a third of ischemic strokes and can cause stroke on a background of potential mechanisms [7,8]. Strokes after HZ infections were initially reported in the early 1970s. Since then, numerous epidemiologic studies have investigated the association between HZ and risk of stroke[9]. VZV is the only recognized human virus that can replicate in cerebral arteries, and it is hypothesized to spread along the nerve fibers to the blood vessels, where it induces further inflammatory and thrombotic responses[10]. Although the short-term and long-term risk of different subtypes of stroke after HV infection have been studied extensively, the results remain controversial. In addition, a quantitative analysis has not been performed to examine the specific association between HZ and stroke risk. Therefore, we conducted a systematic review of the published literature to evaluate the association between HZ and stroke risk.

## Material and methods

The study design was developed and the analyses were conducted following the Preferred Reporting Items for Systematic Reviews and Meta-Analyses (PRISMA) Statement guidelines (S2 Text)[11].

### Literature search

Two authors (Y.L. and Y.Z.) independently performed a systematic search of PubMed and Embase databases for relevant articles written in English from their inception to January 2016. The disagreement were resolved by consulting a third author (RSD). The following search term strategy was used: (transient ischemic attack OR brain infarction OR cerebral infarction OR cerebrovascular diseases OR cerebrovascular disease OR cerebrovascular disorder OR stroke OR ischemic attack OR intracranial embolism OR hemorrhagic stroke) AND (zoster OR shingles OR zona OR herpes zoster).

### Selection criteria

The inclusion criteria were as follows: (1) observational studies (case-controlled or cohort studies) evaluating the risk of HZ and stroke; (2) exposure of interest was HZ; (3) outcome of interest was stroke (ischemic or hemorrhagic); and (4) multivariate-adjusted relative risks (RRs) or hazard ratios (HRs) with a 95% confidence interval (CI) were provided.

### Data extraction and quality assessment

The following data were collected from each of the included studies: name of the first author, year of publication, country where the study was performed, study design (cohort or case controlled), sample size, age of participant at baseline, duration of follow up, number of stroke cases, type of stroke, model (the model that presented the most potentially confounding variables and was adjusted for such errors), adjusted variables, and multivariate-adjusted RRs with 95% CIs. The Newcastle Ottawa Scale (NOS) was adopted to assess the study quality [12]. The included studies were judged based on 3 broad factors: the selection of study populations, the comparability of the populations, and the determination of exposure and outcomes of interest for case-controlled or cohort studies, respectively. This scale assigned a maximum of 9 points for each study. Two independent authors (L.Y. and Y.Z.) performed the data extraction and quality assessment of the included studies. Any disagreement was settled via discussion.

### Statistical analysis

The pooled RR with its corresponding 95% CI was calculated to assess the association of HZ with the risk of stroke. The Q statistic and the *I*^*2*^ statistic were used to assess hHeterogeneity among studies [13]. The *I*^2^ described the percentage of total variation caused by between-study heterogeneity rather than chance [14]. The Dersimonian and Laird random effects model was applied as the pooling method regardless of heterogeneity. All statistical analyses were conducted using STATA 11.0 (Statacorp LP, College Station, TX, USA). All reported probabilities (P values) were 2 sided, and a P value of less than 0.05 was considered statistically significant.

## Results

### Study characteristics

**Fig 1:**
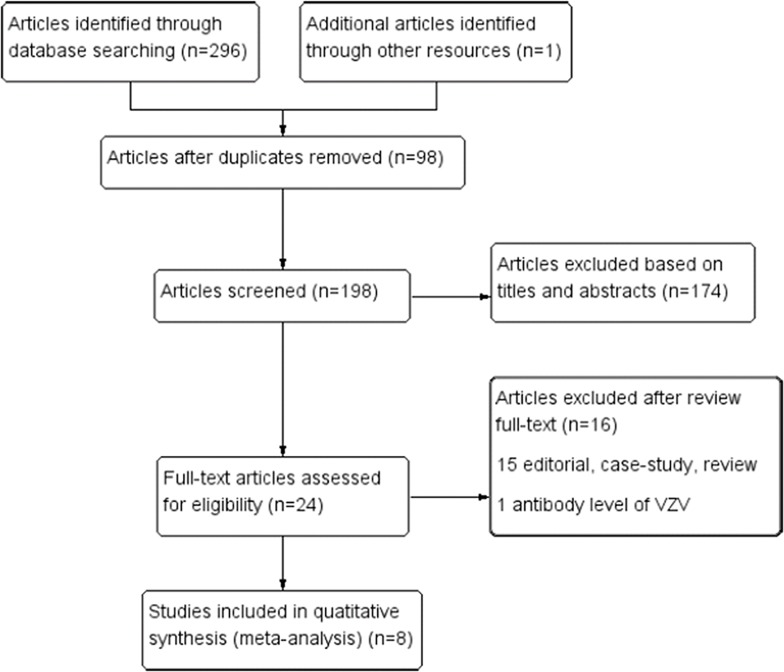
Flow diagram of the selection of studies for the meta-analysis Quantitative synthesis.

The study identification and selection process is summarized in Fig 1. After a screening procedure conducted independently by two reviewers, 8 studies were found to meet the inclusion criteria, and they included 7 studies originally published as full papers [15–21] and one study that was presented as an abstract at a meeting [22]. Of these studies, three were conducted in the UK, two were conducted in Taiwan, one was conducted in the US, one was conducted in Sweden, one was conducted in Denmark, and one was conducted in Germany. The characteristic of the 8 studies are presented in Table 1. All the included studies met the quality criteria, and they ranged from 7 to 8 stars.

**Table 1:** Characteristic of included studies that evaluated the association of herpes zoster and risk of stroke.

Author/year/location	Study design	No. of HZ	No. of Stroke	Type of stroke	Definition of stroke	Assessment of HZ	Adjusted variables	Scores
Minassian1 C, 2015 (UK)[19]	Self-Controlled Case Series	42954	42954	Ischemic stroke	ICD-9-CM	ICD-9-CM	Age	8
Langan SM, 2014 (UK)[15]	Self-controlled case series	6584	6584	Ischemic and hemorrhagic stroke	ICD-10	ICD-10	Age	8
Bricout, 2014 (Germany)[20]	Self-controlled case-series	124462	124462	NA	NA	NA	NA	NA
Yawn BP, 2016 (US)[13]	Cohort Study	4478	2406	NA	ICD-9	ICD-9	Hypertension, dyslipidemia,coronary artery disease, cardiac arrhythmias, congestive heartfailure, diabetes mellitus, vasculopathies andstroke (for MI), depression, and chronicobstructive pulmonary disease	7
Sundström1 K, 2015 (Sweden)[14]	Cohort Study	13296	111	NA	ICD10	ICD10	Age and sex	7
Breuer J, 2014 (UK)[16]	Cohort Study	106601	7979	Ischemic and hemorrhagic stroke	READ codes	READcodes	Sex, age, BMI, smoking status, history of cholesterol>6.2mmol/L, hypertension, diabetes, ischemic heart disease, atrial fibrillation, intermittent arterial claudication, carotidstenosis, and valvular heart disease.	7
Sreenivasan N, 2013 (Denmark)[17]	Cohort Study	117926	4876	NA	ICD-10	ICD-10	Age, sex, and calendar period	8
Kang JH, 2009 (Taiwan)[18]	Cohort Study	7760	745	Ischemic and hemorrhagic stroke	ICD-9	ICD-9	Age, sex, hypertension, diabetes, coronary heart disease, hyperlipidemia, renal disease, atrialfibrillation, heart failure, heart valve/myocardium disease, carotid/peripheral vascular disease, monthly income, urbanization level, andgeographical region.	8

Note: UK: United Kingdom. NA: not available

### Short-term risk of stroke after HZ infection

Four independent studies reported the short-term risk of stroke after HZ infection within 1 month, 3 months and 6 months after HZ infection.

**Fig 2:**
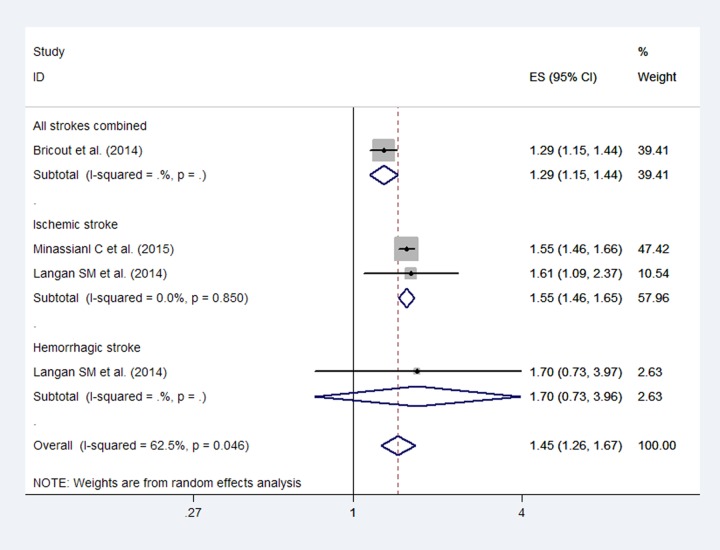
Forest plot based on the included studies indicating the pooled relative risk (RR) of stroke risk during the first 1 month after a herpes zoster infection.

The meta-analysis of the included studies demonstrated a significant association between stroke and HZ in the short term (RR: 1.45; 95% CI, 1.26–1.67; Fig 2). Although high heterogeneity (*I*^2^ = 62.5%) was detected, publication bias was not observed based on Egger’s test (*P* = 0.88). For these studies, the pooled RRs for ischemic stroke and hemorrhagic stroke were 1.55 (95% CI, 1.46–1.65) and 1.70 (95% CI, 0.73–3.96), respectively, during the first 1 month after HZ infection.

**Fig 3:**
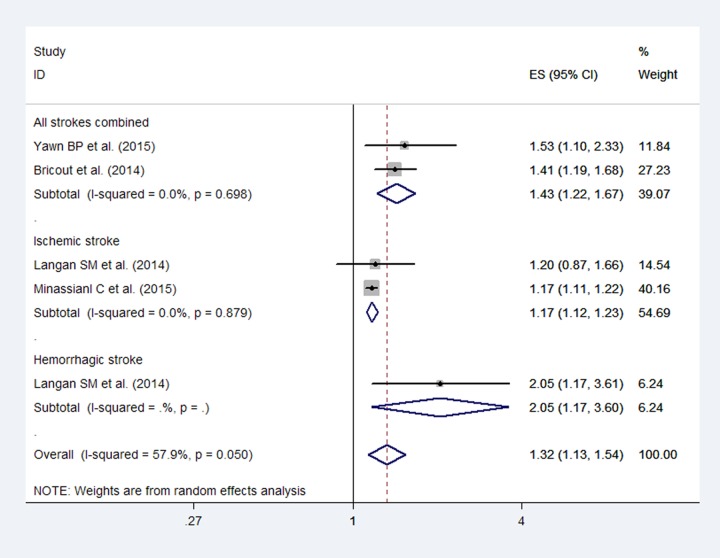
Forest plot based on the included studies indicating the pooled relative risk (RR) of stroke within 3 months after a herpes zoster infection.

After pooling the results, an increased stroke risk was observed (RR: 1.32; 95% CI, 1.13–1.54; Fig 3) within 3 months after herpes zoster, and although moderate heterogeneity was observed (*I*^2^ = 57.9%), publication bias was not detected by Egger’s test (*P* = 0.07). Additionally, the pooled RRs for ischemic stroke and hemorrhagic stroke were 1.17 (95% CI, 1.12–1.23) and 2.05 (95% CI, 1.17–3.60), respectively.

**Fig 4:**
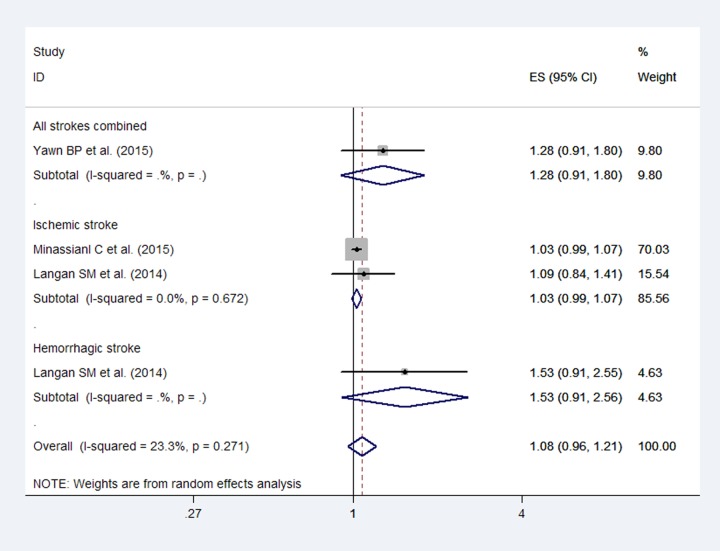
Forest plot based on the included studies indicating the pooled relative risk (RR) for stroke within 6 months after a herpes zoster infection.

The pooled RRs for total stroke were 1.08 (95% CI, 0.96–1.21; Fig 4) within 6 months after a herpes zoster infection, with no heterogeneity (*I*^2^ = 23.3%). An association was not observed between HZ and stroke, and publication bias was not detected by Egger’s test (*P* = 0.07). Additionally, the pooled RRs for ischemic stroke and hemorrhagic stroke were 1.03 (95% CI, 0.99–1.07) and 1.53 (95% CI, 0.91–2.56), respectively.

### Long-term risk of stroke after an HZ infection

The long-term risk assessed in the present study was 1 year and longer than 1 year after an HZ infection.

**Fig 5:**
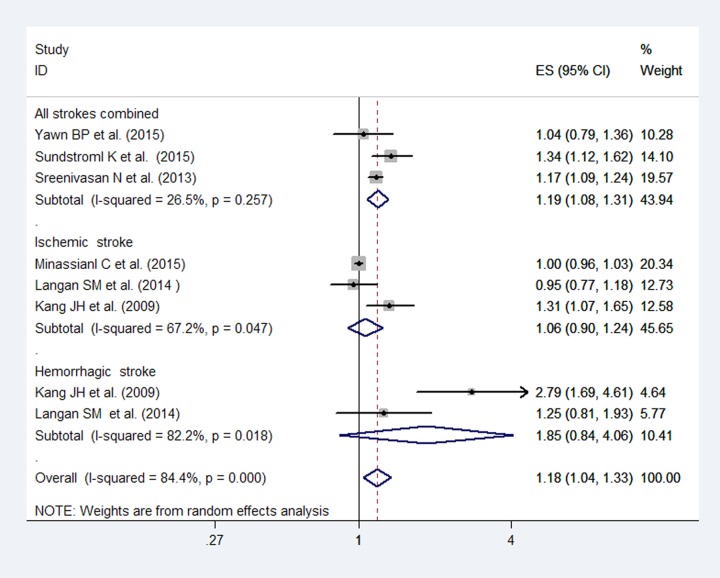
Forest plot based on the included studies indicating the pooled relative risk (RR) for stroke within 1 year after a herpes zoster infection.

Fig 5 shows the results of the 6 enrolled studies that demonstrated an increased risk for stroke within 1 year after an HZ infection (RR: 1.18; 95% CI, 1.04–1.33). However, the pooled RRs were 1.06 (95% CI, 0.90–1.24) and 1.85 (95% CI, 0.84–4.06) for ischemic stroke and hemorrhagic stroke, respectively. Although high heterogeneity (*I*^2^ = 84.4%) was detected, publication bias was not observed based on Egger’s test (*P* = 0.08).

**Fig 6:**
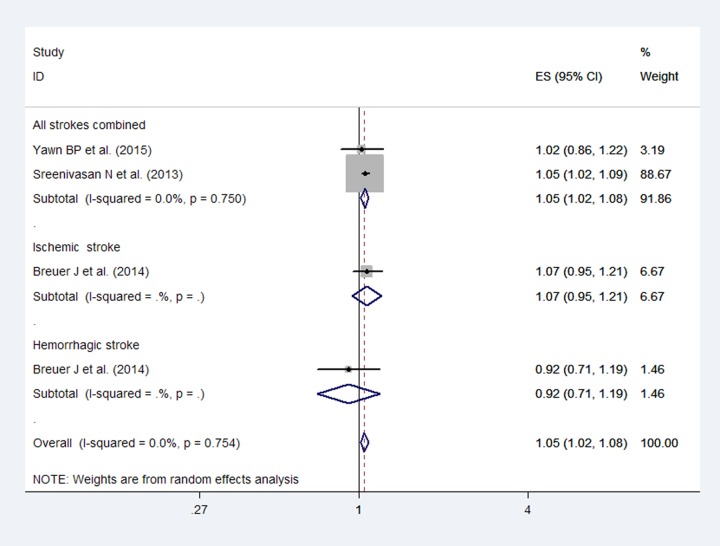
Forest plot based on the included studies indicating the pooled relative risk (RR) for stroke more than 1 year after a herpes zoster infection.

The studies suggested an increased risk of stroke more than 1 year after HZ infection with no heterogeneity (*I*^2^ = 0%; Fig 6). Based on the included studies that provided data involving the stroke subtype, an increase was not observed in the odds of ischemic stroke (RR: 1.07; 95% CI, 0.95–1.21) and hemorrhagic stroke (RR: 0.92; 95% CI, 0.71–1.19) more than 1 year after HZ infection. Publication bias was not detected by Egger's (*P* = 0.40) test.

## Discussion

Our systemic review quantitatively summarized the current literature and analyzed 8 studies to determine the short-term and long-term stroke risk after HZ infection. Overall, the incidence of ischemic stroke is significantly higher in short-term after herpes zoster, whereas a significant relationship was not observed in the long term after HZ infection. With respect to hemorrhagic stroke, herpes zoster correlated positively with the stroke. With respect to hemorrhagic stroke, the association between was not significant except within 3 months after a herpes zoster infection.

We should also pay attention to that nearly 30%-40% of patients with VZV vasculopathy did not have chickenpox or herpes history, but only develop nervous system abnormalities as the onset of symptoms[23]. So that, the correlation between stroke and zoster may be underestimated since VZV can reactivate from the ganglia, travel to directly infect the cerebral vessels in the absence of rash.

Several potential mechanisms may explain the short-term risk for ischemic stroke after HZ infection. First, VZV vasculopathy has been recognized as the leading cause of stroke, latent VZV can be reactivated and spreads along the trigeminal ganglion or other ganglion afferent fibers into the cerebral arteries[24]. VZV is the only human virus which can replicate in cerebral arteries to have been found so far. Previous studies to date suggest that in the patient who was confirmed VZV vasculopathy virologically, a productive viral infection with secondary inflammatory response can lead to pathological vascular remodeling with result of intima proliferation which contributes to vascular obstruction and ischemia of the the cerebral arteries, and with the damage of the media can result in thrombosis, occlusions, infarctions or aneurysms [23,25]. The stroke patients developing after herpes zoster obviously have changes on both brain imaging and angiograms [23,26].Hayman et al reported that the vessel wall of the infarcted brain sections pathology showed vasculitis with lymphocytic infiltration [27].Second, inflammatory cytokines such as interleukin-6 (IL-6) is significantly increased with VZV infections, and the increase is related to arterial thrombosis, although at much lower level[28,29]. Inflammation plays an important role in the etiology of ischemic stroke while hemorrhagic stroke has a completely different etiology (subarachnoid and intracerebral hemorrhagic stroke). In addition, HZ infections are correlated with other major comorbidities, such as diabetes, cardiac disease and hypertension [30–32], which are conditions that have a greater favorable effect on ischemic than hemorrhagic stroke.

The present Meta-Analysis shows an advantage in inclusion of articles with long follow-up durations. Eight studies were included, and most had large populations, involving Americans, Europeans and Asians, thereby strengthening the statistical power of the current study. Furthermore, all the studies in this review were of a cohort design and included self-controlled case series (SCCS) derived from cohort studies [33].Cohort studies can eliminate the possibility of recall and selection bias that can occur in retrospective case-controlled studies. Using the SCCS method, the included studies evaluated the stroke risk following HZ infection within individuals by comparing the risk during exposed periods following HZ infection to the risk during unexposed periods. The major advantage of this study design was that fixed confounders were implicitly controlled for because the analyses were performed within-subject [34]. In the end, since ischemic and hemorrhagic stroke have different pathological changes and etiologies,and some recently published research have data on stroke subtype, so data from the ischemic and hemorrhagic stroke groups were analyzed separately. And short-term and long-term influence of VZV infections on stroke risk were also assessed in this current study.

Certain limitations should also be acknowledged when interpreting the results from this study. Our study is a meta-analysis of included observational studies,it is vulnerable to bias introduced by methodological and clinical heterogeneity in the primary studies.First, because of the observational nature of the included studies, loss to follow up was evitable. Second, due to limited data, we were unable to analyse possible significant differences in the associations by conducting subgroup analyses. Finally, the observed association between HZ and stroke risk may have been affected by unevaluated or residual confounding factors. Persons with HZ may have diabetes, cardiac disease and hypertension. Most of the studies included in the meta-analysis but not all adjusted for these and other potential confounders.

Findings from previous studies showed that VZV vaccination can reduce incidence of HZ and post-herpetic neuralgia in the elderly population [35,36]. Minassian et al. [21] reported there has no difference in the risk between zoster diagnosis vaccinated and unvaccinated patients during the following first month for ischemic stroke, which was primarily based on the low uptake of the HZ vaccine among the study participants, only 3% developed HZ infection after vaccination, thus it is hard to draw conclusions whether vaccination can affect the correlation of HZ and acute cardiovascular events. As previous studies have found in adults above 50 years HZ vaccination may be a worthwhile intervention to reduce the risks of herpes zoster and neuralgia[37,38]. The vast majority of studies showed vaccination to be cost-effective, but for persons aged 50 years, it does not seem to represent good value[39]. Hence, further study is needed because of low vaccination rates, and more efforts are required to improve vaccination in the routine care of elderly population. Langan et al. proposed that stroke risk after HZ can be reduced through treatment of antiviral therapy [17].It is generally known that antiviral drugs showed efficacy for relieving acute pain, reducing zoster severity and accelerating healing. Therefore, it follows that as antiviral medicine are known to reduce inflammation,the drug may have the prevention of other postzoster complications, including vascular events, by reducing inflammation [24,40].

### Conclusions

In conclusion, this systemic review and meta-analysis provided strong evidence that HZ infection is one obvious short-term risk factor for ischemic stroke. Because of the high prevalence and incidence of HZ infection and stroke in the general population, the observed association between HZ infection and stroke has clinical and public health importance. Evidence of HZ infection increasing the risk of hemorrhagic stroke was not observed. However, additional studies are required to explore the underlying mechanisms and intervention approaches for a population of patients with herpes zoster affected.

## Supporting information

S1 Text Search strategy used to identify theincluded studies. (DOCX)

S2 Text PRISMAChecklist. (DOC)

S1 Table Data used for meta-analysis. (XLSX)
