# Supplementary material for: Herpes zoster and the risk of ischemic and hemorrhagic stroke: A systematic review and meta-analysis
Source: PLoS One. 2017 Feb 8;12(2):e0171182. doi: 10.1371/journal.pone.0171182 (PMC5298244; doi:10.1371/journal.pone.0171182)
Supplement: S1 Text — (DOCX) [file pone.0171182.s001.docx]

S1 Text. Search strategy used to identify the included studies in Pubmed.

((((((((((transient ischemic attack[Title/Abstract]) OR brain infarction[Title/Abstract]) OR cerebral infarction[Title/Abstract]) OR cerebrovascular diseases[Title/Abstract]) OR cerebrovascular disease[Title/Abstract]) OR cerebrovascular disorder[Title/Abstract]) OR stroke[Title/Abstract]) OR Ischemic Attack[Title/Abstract]) OR Intracranial Embolism[Title/Abstract])) AND ((((Zoster[Title/Abstract]) OR shingles[Title/Abstract]) OR zona[Title/Abstract]) OR Herpes Zoster[Title/Abstract])
